# Endogenous cueing effects for detection can be accounted for by a decision model of selective attention

**DOI:** 10.3758/s13423-019-01698-3

**Published:** 2020-01-06

**Authors:** Miranda L. Johnson, John Palmer, Cathleen M. Moore, Geoffrey M. Boynton

**Affiliations:** 1 Department of Psychology, University of Washington, Seattle, WA 98195 USA; 2 Department of Psychology, University of Iowa, Iowa City, IA USA

**Keywords:** Attention, Selective attention, Partially-valid cueing, Visual attention

## Abstract

Spatial cues help participants detect a visual target when it appears at the cued location. One hypothesis for this cueing effect, called *selective perception*, is that cueing a location enhances perceptual encoding at that location. Another hypothesis, called *selective decision*, is that the cue has no effect on perception, but instead provides prior information that facilitates decision-making. We distinguished these hypotheses by comparing a simultaneous display with two spatial locations to sequential displays with two temporal intervals. The simultaneous condition had a partially valid *spatial* cue, and the sequential condition had a partially valid *temporal* cue. Selective perception predicts no cueing effect for sequential displays given there is enough time to switch attention. In contrast, selective decision predicts cueing effects for sequential displays regardless of time. We used endogenous cueing of a detection-like coarse orientation discrimination task with clear displays (no external noise or postmasks). Results showed cueing effects for the sequential condition, supporting a decision account of selective attention for endogenous cueing of detection-like tasks.

Whether reading a book or having a conversation in a crowded room, selective attention allows one to select relevant information while limiting distractions. One way to measure selective attention is *partially valid cueing* (Posner, Snyder & Davidson, 1980), in which a cue indicates where a target stimulus is most likely to appear. For some proportion of trials, the cue is valid and the target appears at the cued location. For a smaller proportion of trials, the cue is invalid and the target appears at an uncued location. Targets are more likely to be detected at the cued location.

What causes such *spatial cueing effects*? One hypothesis, *selective perception,* posits that perceptual encoding is enhanced for information at the cued location. Under this hypothesis, the selective processing is often described using a spotlight metaphor (Posner et al., 1980), or a limited resource (Norman & Bobrow, 1975). It is assumed that, given time, one can switch selective processing to different spatial locations. This idea has been generalized to allow allocation of renewable resources over space and time (Denison, Heeger, & Carrasco, 2017).

A second hypothesis, *selective decision*, posits that perceptual encoding has unlimited capacity, and therefore encoding is the same at cued and uncued locations. Rather than enhancing perceptual encoding, the cue is used in decision-making to increasingly weight the cued information. This hypothesis is a generalization of Bayesian decision-making in that the cue provides a prior that influences how information for the different locations is used in decision. An early example of this hypothesis is the weighting model of Kinchla, Chen, and Evert (1995). For an optimal Bayesian model and a detailed development of this hypothesis, see Shimozaki, Eckstein, and Abbey (2003). For this hypothesis, attentional switching is irrelevant because perception has unlimited capacity and thus cannot be improved by selective processing of the stimulus.

In the literature, some studies support selective perception (Dosher & Lu, 2000; Posner et al., 1980), and others support selective decision (Kinchla et al., 1995; Shimozaki, et al., 2003). One recent hypothesis is that selective decision mediates endogenous cueing with clear displays and selective perception contributes to both exogenous and endogenous cuing of displays with external noise or postmasks (Dosher & Lu, 2000; Smith, 2000). In this article, we focus on endogenous cueing and clear displays, which appear most likely to be mediated by selective decision.

Our aim is to distinguish these hypotheses by comparing simultaneous and sequential displays (Eriksen & Spencer, 1969; Shiffrin & Gardner, 1972). This paradigm manipulates the amount of perceptual information that must be encoded within a given time interval, while keeping the decision component of the task constant. In their groundbreaking study, Shiffrin and Gardner (1972) tested whether the detection of letters is limited or unlimited in capacity. Participants detected a target letter among distractor letters that were shown either simultaneously or sequentially. A limited-capacity model predicts better performance for sequential compared with simultaneous displays. An unlimited-capacity model predicts equal performance for simultaneous and sequential displays. They found equal performance in the simultaneous and sequential conditions, which is consistent with letters being processed with unlimited capacity.

The simultaneous–sequential paradigm has since been used to test capacity limits for a variety of stimuli. The results support unlimited-capacity processing for simple stimuli, such as simple features, alphanumeric digits, and simple surface completion, but limited-capacity processing for more complex stimuli, such as words and objects (Attarha, Moore, Scharff, & Palmer, 2014; Duncan, 1980; Huang & Pashler, 2005; Pashler & Badgio, 1987; Scharff, Palmer, & Moore, 2011, 2013).

Key to distinguishing limited and unlimited capacity is the assumption that participants can shift attention with sequential displays. To pursue this, Duncan, Ward, and Shapiro (1994) measured *attentional dwell time*: the time needed to completely shift attention from one object to another. The identification of the stimulus in the second display depended on the stimulus onset asynchrony (SOA), which indicates that attending to the first stimulus interfered with identificiation of the second stimulus. Across several studies, estimates of the mean dwell times vary from 150 to 600 ms (Moore, Egeth, Berglan, & Luck, 1996; Petersen, Kyllingsbæk, & Bundesen, 2012). Thus, an SOA of about 1,000 ms is sufficient to switch attention on almost all trials.

In the current study, we used the simultaneous–sequential paradigm to distinguish the predictions of selective perception and selective decision. Comparisons of spatial and temporal cueing have not often been made (Rohenkohl, Gould, Pessoa, & Nobre, 2014). We focused on conditions with clear displays (no external noise or postmasks), and endogenous cues. Stimuli were presented in one of two temporal intervals. In the simultaneous condition, a partially valid cue indicated the most likely spatial location of a stimulus. In the sequential condition, a partially valid cue indicated the most likely temporal interval. Both hypotheses predict a cueing effect in the simultaneous condition. Selective decision also predicts a cueing effect in the sequential condition because the cue is used as a prior for decision-making. In contrast, selective perception predicts no cueing effect in the sequential condition given there is time to switch attention from one interval to the other.

## Experiment

### Method

#### Overview

**Fig. 1:**
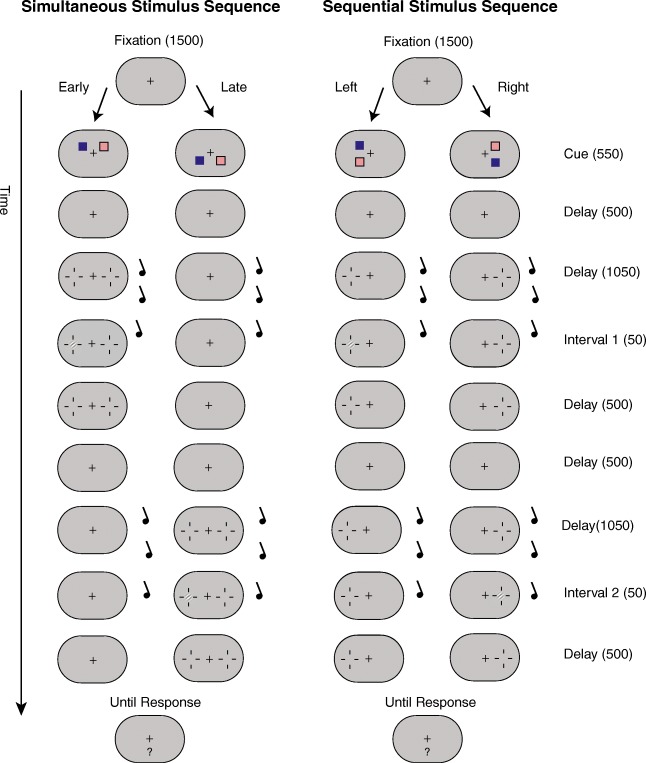
Schematic of the trial sequence for the simultaneous and sequential conditions (not to scale). Display durations are shown in milliseconds. In the simultaneous condition, the cue was spatial, indicating which side of fixation the target was most likely to appear. In the sequential condition, the cue was temporal, indicating which stimulus interval was most likely to contain the target. In the example sequence, the cue color was blue, and valid trials are shown with a right-leaning Gabor target. The red cue is shown here with an outline to make it more distinct for reprinting

A single brief Gabor patch was presented that was either tilted left or right, and the task was to indicate whether the tilt was to the left or the right. Such a coarse orientation discrimination yields the same performance as detection (Thomas & Gille, 1979). Hence, we consider it a detection-like task. This stimulus was presented to either the left or right of fixation and in one of two possible intervals (see Fig. 1). In the simultaneous condition, the stimulus was presented at one of two locations in a known interval. In the sequential condition, the stimulus was presented in one of two intervals at a known location.

#### Participants

There were 13 paid participants including one author (M.J.). All participants had normal or corrected-to-normal acuity. All gave written and informed consent in accord with the human subjects Institutional Review Board at the University of Washington, in adherence with the Declaration of Helsinki.

To determine the number of participants, we used pilot data from a previous partially valid cueing experiment. Participants (*N* = 12) each completed a coarse orientation discrimination experiment with similar methods as the simultaneous condition of the present experiment. We observed a cueing effect of 7 ± 1%. Seeking this size of cueing effect, a power analysis suggested a minimum of seven participants. To be conservative, we decided to use a minimum of 12. Due to accidents of scheduling, we tested a total of 13.

#### Apparatus

Displays were presented on a linearized CRT monitor (Sony GDM-FW900) with resolution 1,024 × 640 pixels refreshing at 120 Hz. The monitor was viewed from 60 cm and had a mean luminance of 56 cd/m^2^. Stimuli were created with MATLAB (MathWorks, Natick, MA) and The Psychophysics Toolbox (Brainard, 1997). Gaze position was monitored for all trials using an EyeLink 1000 (SR Research). Trials containing blinks or broken fixation were excluded from analysis. Such excluded trials were infrequent and occurred on only 3 ± 0.5% of trials across participants.

#### Stimuli

Participants judged the orientation of a Gabor patch that had a spatial frequency for the grating component of four cycles per degree, a standard deviation of the Gaussian envelope of 0.5 degrees and was truncated to be 3 degrees in diameter. The Gabor was in one of two orientations: 130 degrees (left-tilting) or 40 degrees (right-tilting). It was presented 10 degrees to the left or right of fixation. The contrast of the Gabor was adjusted for each participant such that their performance with a valid cue was between 70%–80% correct for both simultaneous and sequential trials. The mean contrast used was 8%.

#### Procedure

The simultaneous and sequential conditions are shown schematically in Fig. 1. In the simultaneous condition, the cue indicated both the most likely target location and, with certainty, the temporal interval. For *early-interval blocks*, the trials began with fixation for 1,500 ms, followed by the cue for 550 ms. The cue consisted of one red and one blue square, each 0.75 degrees in width and height. Both squares were one degree above fixation, and one degree to the left and right of fixation. Participants were assigned a cue color of red or blue. They were told that the cue indicated which side of fixation the target was most likely to appear, and the position of the cue above fixation indicates an early-interval trial. The random assignment of color was to ensure the cue was fully endogenous. The probability of the target appearing at the cued location was .8, and for the uncued location was .2.

Following the cue was a 500-ms delay with only the fixation cross. To reduce spatial uncertainty, there was a second 1,050-ms delay with fiducial markers that indicated the locations where the target could appear. Additionally, to reduce temporal uncertainty, there was a sequence of three tones. During the time before the stimulus, two 500 Hz tones were played for 250 ms each, with a 250-ms break following each tone.

After the delay, the stimulus was presented for 50 ms and a single 750 Hz tone was played for 250 ms. Following the stimulus was a 500 ms delay with fiducial markers. This continued display of the fiducial markers was to avoid an offset of the marker near in time to the presentation of the Gabor. An additional 500-ms delay then occurred without the fiducial markers. There was then a second 1,050-ms delay during which two 500 Hz tones were played as before. Following the 1,050-ms delay was a 50-ms blank interval, during which a single 750 Hz tone was played for 250 ms. There was a final 500-ms delay with only the fixation cross, after which participants were prompted to respond, and given unlimited time to do so. Reaction time was not recorded.

The trial sequence for *late-interval blocks* was similar to that of early-interval blocks. The differences were that the first interval was blank with no stimulus or fiducial markers, and instead the second interval contained the stimulus and fiducial markers. Additionally, the cue appeared below fixation to indicate a late-interval trial.

In the sequential condition, the cue indicated when the target was most likely to appear and with certainty indicated the location. The trial began with a fixation cross for 1,500 ms, followed by a cue for 550 ms that consisted of a red and a blue square either one degree to the left or one degree to the right of fixation to indicate where the target would appear. The squares were positioned vertically such that one square appeared one degree above fixation, and the other square appeared one degree below fixation. The vertical location of the cue indicated which interval the target was most likely to appear. Specifically, a cue above fixation indicated that the target had an 80% chance to appear in the first interval, and a cue below fixation indicated that the target had an 80% chance to appear in the second interval.

Following the cue was a 500-ms delay with only the fixation cross. There was then a 1,050-ms delay with a fiducial marker, and two 500 Hz tones were played as before. The first interval was then shown for 50 ms, and a single 750 Hz tone was played for 250 ms. The first interval was followed by a 500-ms delay with the fiducial markers. An additional 500-ms delay then occurred without the fiducial markers. There was then a second 1,050-ms delay during which the fiducial marker was displayed and two 500 Hz tones were played as before. The second interval was then shown for 50 ms, accompanied by a 750 Hz tone for 250 ms. Following the second interval was a 500-ms delay with the fiducial marker. Participants were then prompted to respond, and given unlimited time to do so. The total SOA between the onset of the intervals was 2,100 ms.

Prior to the experiment, participants completed two to three training sessions during which they learned to use the cues and perform the task. Participants then completed 25 experimental sessions. Each session consisted of eight randomly ordered blocks of 20 trials, making 160 trials per session and 4,000 trials per participant.

#### Predictions

According to selective perception, perceptual encoding involves a limited resource that can be selectively allocated in space. This resource can be switched between locations, and is renewable over time. For brief simultaneous displays, there is no time to switch attention, so participants have only one chance to allocate resources. The cued location is more likely to contain the target; therefore, it helps to allocate resources to that location. In contrast, in the sequential condition the location of the target is known, and the cue indicates the temporal interval that is likely to contain the target. Given a long enough SOA, participants can switch attention and allocate resources to both the cued and uncued intervals. Under these conditions, the selective perception hypothesis predicts no advantage for valid over invalid cues.

According to selective decision, perceptual encoding is unlimited in capacity: The quality of perceptual encoding cannot be enhanced by selective attention. Instead, cueing effects arise because the cue is used to improve decision-making. In both the simultaneous and sequential conditions, participants weight information at the cued location more heavily than information at the uncued location. Therefore, the selective decision hypothesis predicts a cueing effect for both conditions.

## Results

**Fig. 2:**
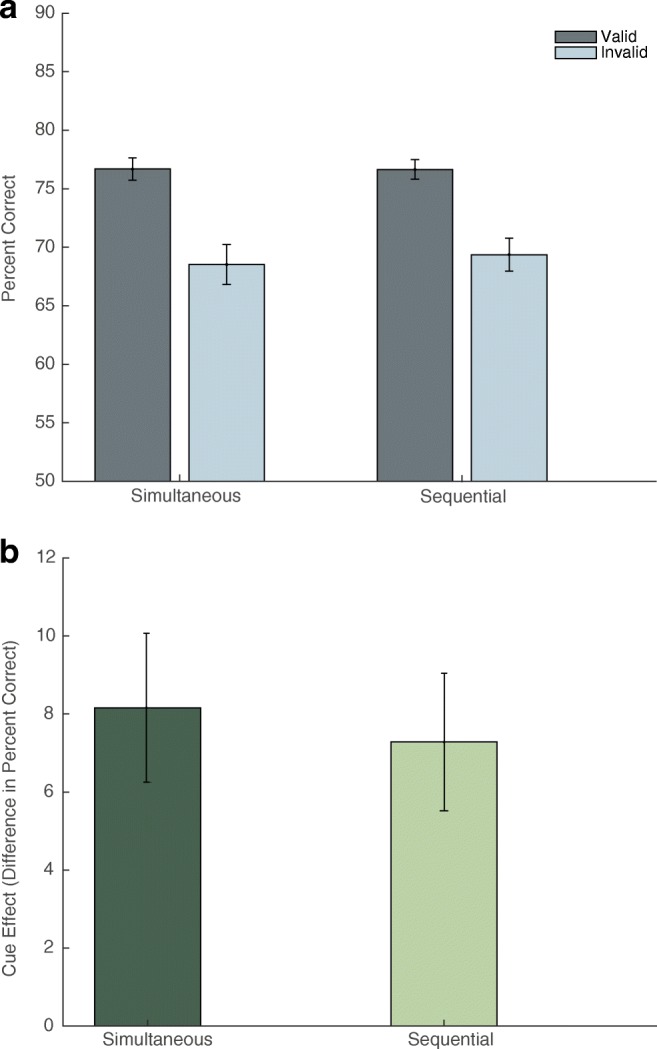
**a** Percent correct for valid and invalid cues, for both the simultaneous and sequential conditions. Error bars represent standard errors. **b** The cueing effect for the simultaneous and sequential conditions, which is calculated as the difference in performance when the cue was valid versus when it was invalid

In Fig. 2a, percentage correct is shown for both the valid and invalid cue conditions, and for both simultaneous and sequential displays. A repeated-measures ANOVA was used with stimulus condition (simultaneous/sequential) and cue condition (valid/invalid) as factors. Performance was better when the cue was valid compared with invalid, *F*(1, 12) = 20.23, *p* < .001. There was no effect of stimulus condition, *F*(1, 12) = 0.31, *p* = .59, and no interaction, *F*(1, 12) = 0.44, *p* = .52.

For the simultaneous condition, average performance was 76.7 ± 1% for the valid condition, and 68.5 ± 2% for the invalid condition. As shown in Fig. 2b, the mean difference between the valid and invalid conditions was 8 ± 2% and was reliable, *t*(12) = 4.27, 95% CI [3.9, 12.3]. For the sequential condition, average performance was 76.6 ± 1% for the valid condition, and 69.4 ± 1% for the invalid condition. The mean difference was 7 ± 2% and was reliable, *t*(12) = 4.13, 95% CI [3.4, 11.1].

## General discussion

### Summary of results

Selective perception and selective decision can both account for typical partially valid cueing effects. To distinguish them, we used the simultaneous–sequential paradigm in which stimuli in the high-probability and low-probability locations were either presented simultaneously within one interval, or sequentially across two intervals. The SOA for the sequential condition was long enough to make it likely that participants could switch attention on all trials. We found similar cueing effects for simultaneous and sequential conditions. This result is inconsistent with selective perception, which posits the cueing effect is due to limited capacity in encoding. Instead, it is consistent with selective decision, which posits the cueing effect is due to using cue information in decision-making.

### Relation to previous research on temporal cueing

Consider next the literature on temporal cueing. We focus on studies with SOAs of 500 ms or more because both selective perception and selective decision predict cueing effects for short SOAs because there is not enough time to switch attention (for general reviews, see Correa, Lupiáñez, Madrid, & Tudela, 2006; Rolke & Ulrich, 2012; Shimozaki, Schoonveld, & Eckstein, 2012).

In an early temporal cueing study, Coull and Nobre (1998; see also Griffin, Miniussi, & Nobre, 2001) used a character discrimination task, and their most relevant conditions had SOAs of 0.3 and 1.5 s. The magnitude of the cueing effects declined sharply with SOA. In another study, Denison, Carrasco, & Heeger (2019) measured temporal cueing using fine orientation discrimination among Gabor patches and a postcue. They found near-zero cueing effects for a SOA of 800 ms, and cueing effects of up to 0.5 *d*′ units for short SOAs. Both of these results are consistent with selective perception because the cue effect declined with SOA.

Other studies have found results consistent with selective decision. Kinchla et al. (1995) had participants detect a target among four sequentially presented letters with an SOA of 1,500 ms. Participants were better at detecting the target when it appeared in the cued interval than when it appeared in the uncued interval, which is consistent with selective decision. Correa, Lupiáñez, and Tudela (2005) measured temporal cueing in a rapid serial visual presentation (RSVP) sequence using letter detection with SOAs of 414 and 1,057 ms. For the long SOA, they found a cueing effect of about 0.4 *d*′ units. These effects were similar to what was found for the short SOA, which is consistent with selective decision.

Why might there be such divergent results? An interesting explanation is that different tasks have different capacity limits on the processing of multiple stimuli. For example, simple detection and word categorization have been shown to have different capacity limits (Pashler & Badgio, 1987; Scharff et al., 2011). These findings, taken together with the results of the current study, are consistent with selective perception for more complex tasks, and selective decision for simple detection.

Another explanation is that temporal uncertainty might have varied with SOA. Decreases in temporal uncertainty increase accuracy (Lasley & Cohn, 1981) and decrease response time (Niemi & Näätänen, 1981). Furthermore, some have shown that decreases in temporal uncertainty decrease cueing effects (Gould, Wolfgang, & Smith, 2007). In our experiments, a sequence of warning tones and fiducial markers minimized temporal and spatial uncertainty. Our pilot studies indicated that minimizing uncertainty was necessary to prevent changes in performance as a function of SOA.

The present study has larger implications. A common view is that selective perception is sufficient to account for all effects of partially valid cueing (e.g., Denison, Heeger, et al., 2017; Nobre & Ede, 2017). Based on our results, and the results we have cited in the literature, we argue that selective perception alone can be rejected. Instead, theories are needed that include a role for both selective decision and selective perception.

### Conclusion

The simultaneous–sequential paradigm was adapted to distinguish two hypotheses for selective attention: selective perception versus selective decision. For a detection-like coarse orientation discrimination task with endogenous cues and clear displays, the results were consistent with selective decision and not selective perception. Other studies using different tasks have found contrary results. While the differences among these studies need to be sorted out, we argue that one can reject selective perception as the universal account of partially valid cueing.
